# Cervical cancer screening practices and its associated factors among females of reproductive age in Durame town, Southern Ethiopia

**DOI:** 10.1371/journal.pone.0279870

**Published:** 2022-12-30

**Authors:** Girma Amado, Fitsum Weldegebreal, Simon Birhanu, Yadeta Dessie

**Affiliations:** 1 Durame General Hospital, Kembata Tembaro, Southern Nation National People Region, Harar, Ethiopia; 2 Department of Medical Laboratory Science, College of Health and Medical Sciences, Haramaya University, Harar, Ethiopia; 3 School of Nursing and Midwifery, College of Health and Medical Sciences, Haramaya University, Harar, Ethiopia; 4 School of Public Health, College of Health and Medical Sciences, Haramaya University, Harar, Ethiopia; Arba Minch University, ETHIOPIA

## Abstract

**Background:**

An estimated 22 million Ethiopian women between the ages of 15 and 49 are affected by cervical cancer each year, with 7095 cases and 4732 fatalities. Cervical cancer screening is one of the prevention methods, although Ethiopia has a low coverage rate. Furthermore, data on the use of cervical cancer screening services in the country is scarce. Therefore, we aimed to assess cervical cancer screening practices and its associated factors among females of reproductive age in Durame, Southern Ethiopia.

**Methods:**

A community-based cross-sectional study was conducted using a multi-stage sampling technique among 460 females of reproductive age from March to April 2020. Data were collected using interviewer-administered questionnaires and analyzed using the Statistical Package for Social Science (SPSS) Version 20. Bivariable and multivariable logistic regressions were carried out to determine the association between independent and dependent variables. The adjusted odds ratio (AOR) with a 95% confidence interval (CI) and a P-value < 0.05 were used to declare the statistical association.

**Results:**

We found that cervical cancer screening practice in this study was 13.8% [95% CI:(10.4–17.2)]. Having a positive attitude [AOR = 5.2, 95% CI:(1.4, 20.0)], having a good knowledge [AOR = 5.4, 95% CI:(1.5,19.5)], being informed about cervical cancer by health professionals [AOR = 3.5, 95% CI:(1.3,9.8)], average monthly income greater than 3000 *Ethiopian Birr* (ETB) [AOR = 4.9, 95% CI:(1.1, 22)], and having a history of sexually transmitted infections [AOR = 4.2, 95% CI:(1.4,12.85)] were the factors associated with cervical cancer screening practice.

**Conclusions:**

The practice of cervical cancer screening was found to be very low, being influenced by women’s attitudes, knowledge, having health professionals as sources of information, monthly income, and history of sexually transmitted infections. Thus, it is necessary to increase awareness and knowledge about cervical cancer and improve attitudes toward cervical screening services to improve the uptake of the screening. Health professionals also have to play a pivotal role in properly addressing information about cervical cancer.

## Background

Cervical cancer is cancer that occur on the cervix, mostly caused by Human Papilloma Virus (HPV subtypes 16 and 18), transmitted through sexual contacts [1]. It is known to be the fourth most frequent cancer in women, representing 6.6% of all female cancers [2]. More than 85% of deaths from cervical cancer occur in low and middle-income countries [2]. Evidence have shown that in Sub-Saharan Africa, there were 111632 new cases of cervical cancer in 2018 of which 68% died [3].

Cervical cancer affects an estimated 22 million women (over the age of 15) worldwide, accounting for 25.8% to 32% of all female cancers. Each year, 6294 women are diagnosed with cervical cancer, with 4884 dying as a result [4]. Cervical cancer is the most common cancer in Ethiopia, accounting for 13.4% of all malignancies, and women account for around two-thirds of all cancer deaths [5]. Only 0.6% of Ethiopian women aged 18 to 69 were screened for cervical cancer [5].

Cervical cancer progresses slowly from the precancerous stage to invasive cancer, and it can be prevented through screening for early detection, which gives an opportunity for early treatment [3, 6, 7]. The majority of cervical cancers in Sub-Saharan Africa (over 80%) are diagnosed late, owing to a lack of information about cervical cancer screening and access to cervical cancer screening services. As a result, survival rates following surgery or radiotherapy treatment are low [6, 7].

Cervical cancer screening is the asymptomatic detection of pre-cancer and cancer in women at risk, with at least one screening suggested for women aged 30–49 years [1]. According to the World Health Survey (2015), 19% of eligible women in developing countries and 63% of those in developed nations had their cervical cancer screening done using a Pap smear or visual inspection with acetic acid (VIA) [6]. It is estimated that in developing countries, around 60% of the women who are diagnosed with invasive cervical cancer have never had cervical screening [1, 5, 8].

Studies have shown that knowledge about cervical cancer [8], women’s level of education [9, 10], number of sexual partners [10], awareness about the available cervical cancer screening methods [9, 11], gender of the service provider [11], privacy [11], age [12], history of STI [13], attitudes of women towards pre-cervical cancer screening [13], family planning utilization [14], and cultural and religious beliefs [11] were hindering factors of cervical cancer screening utilization.

According to the Ethiopian Federal Ministry of Health (EFMOH) report, the EFMOH and Pathfinder worked together to pilot VIA screening along with access to cryotherapy in Ethiopia in 2009. Then, based on the findings from this pilot, the EFMOH scaled up the service. The ministry also developed comprehensive cervical cancer prevention and control guidelines, along with VIA and cryotherapy training materials, in 2015. Despite this, there is limited contextual evidence on cervical cancer screening practice in Ethiopia [13]. Therefore, this study was aimed at assessing cervical cancer screening practices and associated factors among females of reproductive age in Durame town, Southern Ethiopia, to provide baseline information.

## Materials and methods

### Study setting

The study was conducted in Durame town, Kembata Tembaro Zone, southern Ethiopia. Durame town is located 280 km from Addis Ababa, which is the capital of Ethiopia. According to the Durame town Health Bureau, the town has three *kebeles (the smallest administrative unit)*, namely Lalo, Kasha, and Zeraro, with a total population of 50213 and 10248 households. Of these, 24604(49%) were males, and 26165(51%) were females. An estimated number of females of reproductive age (15–49 years) was 11700. In the town, there was one general hospital, one health post, three health centers, seven private clinics, and eleven pharmacies. A facility in the town called *"Dr*. *Bogalech Gebure Memorial General Hospital"* has routinely offered VIA-based cervical cancer screening services.

### Study design and period

A community-based, cross-sectional study was conducted from March 2020 to April 2020.

### Population

Females of reproductive age (15–49 years) who had lived in Durame town for six months as a resident before the data collection period were eligible for the study. Females who were unable to give information during the study time were excluded from the study.

### Sample size determination

Sample size was calculated using a single population proportion formula for the first objective and a power approach using a double proportion formula for the second objective. Using a single population proportion formula, the sample size calculated as 269 by taking the proportion of cervical cancer screened (p) = 19.6% [23] and adding 10% non-response rate. Using the double proportion formula, by taking significantly associated factors (age, history of STI, positive attitude of women, family planning utilization) from previous studies [12–14] and using the Stat-calc of Epi Info statistical software Version 7, we got a sample size of 460. Then, by comparing the two calculated sample sizes, we took the larger sample size (460) as the final sample size.

### Sampling procedure

A multistage sampling technique was applied to select the required study participants. There are three kebeles*(the smallest administrative unit)* found in the town. From those three *kebeles*, two *kebeles* (Lalo kebele and Kasha kebele) were selected using a simple random sampling technique. Then, the total sample size was proportionally allocated to those selected *kebeles*. Using a family folder, which was prepared and regularly updated by the health extension workers, as a sampling frame, a systematic sampling technique was applied to select the households from the family folder after calculating the sampling interval (K^th^-value). The calculated k^th^ interval was 10 (k^th^ = 4694/460), and the first household (random start) was chosen using a simple random sampling method (lottery method) from those ten households found in the first interval. Then, in every tenth household, a female of reproductive age was recruited. If no female of reproductive age was found in the selected house/vicinity, we were shifted to the next nearby household, and the interval was continued to the next K^th^ value.

### Data collection procedures and tools

A pre-tested structured questionnaire adapted from different literature [15–18] was used for data collection. The questionnaire contains questions about socio-demographic characteristics, knowledge, attitude, information (invitation) by the health care provider, sexual and reproductive health characteristics, and cervical cancer screening practice. The data were collected by twelve nurses and two supervisors. A tool prepared in local languages (Amharic and Kembatigna) was used to collect data.

### Operational definitions

#### Cervical cancer screening practice

The proportion of women who have ever been screened for pre-cervical cancer at least once in life time [13, 19].

#### Good knowledge

Refers to those who have scored greater than or equal to the mean value regarding knowledge assessment questions [12, 20].

#### Positive attitude

Refers to those who scored the mean and above regarding attitude questions [20–22].

### Data quality control

The questionnaire was prepared in English and then translated into Amharic and Kembatigna *(the local language)*. Then a back translation to English was made by language experts to check its consistency. Data collectors and supervisors were trained for two days, addressing the aim of the study, data collection tools, sampling procedures, and data collection procedures. We conducted a pre-test on 5% of the total sample size in one of Mudula town’s *kebele*, which is close to the current study location. All required amendments were made based on the feedback from the pretest. The completeness and consistency of each questionnaire were checked daily by the supervisors and principal investigator. Likewise, the double data entry method was employed to avoid data entry errors.

### Data analysis

The collected data was checked for its completeness and consistency, coded, and entered into Epidata version 3.1, and exported to SPSS version 20 Statistical Software for cleaning and analysis. The mean, median, standard deviation, frequency, and percentages were used to summarize the findings. A bivariable logistic regression analysis was computed to test whether dependent and independent variables had an association. Variables with a P-value of <0.25 in the bivariable logistic regression analysis were included in the multivariable analysis. Multicollinearity was checked by a variance inflation factor (VIF) or tolerance. The Hosmer-Lemeshow goodness of fit was used to check the model’s fitness. The adjusted odds ratio with a 95% confidence interval was used to assess the strength of the association between dependent and independent variables at a P-value of <0.05 significance level.

### Ethical approval and consent to participate

The ethical approval was obtained from Haramaya University, College of Health and Medical Sciences, Institutional Health Research Ethics Review Committee (IHRERC) with a reference number of IRERC/030/2020. First, permission and consent were obtained from the Southern Nation Nationalities People Region (SNNPR) health bureau, the Kembata Tembaro zone health department, and the Durame health office. Then, a signed written consent was sent from the department and submitted to the head of the health facilities and local authorities. Participants were informed clearly about the purpose of the study, and informed, written, voluntary, and signed consent was obtained from them. This study was conducted in accordance with the Declaration of Helsinki.

## Results

### Socio-demographic characteristics

A total of 442 respondents were interviewed out of 460 approached, resulting in a 96% response rate. The mean age of the respondents was 27 years, with an 8-year standard deviation (SD). The majority of the respondents, 328(74.2%), were Kembata in their ethnicity, and 241(54.5%) had completed primary and secondary level education. More than half 232(52.5%) of the respondents were married, and 313(70.8%) had less than 1000 *Ethiopian Birr* (ETB) in average monthly income (Table 1).

**Table 1:** Socio-demographic characteristics of females (15–49 years) in Durame town, SNNPR, Ethiopia, 2020 (n = 442).

Variables	Category	Frequency	Percent
Age	15–24 years	145	32.8
	25–34 years	187	42.3
	35–49 years	110	24.9
Ethnicity	Kembata	328	74.2
	Tembaro	74	16.8
	Hadiya	24	5.4
	Wolayta	11	2.5
	Others*	5	1.1
Religion	Protestant	242	54.8
	Orthodox	104	23.5
	Catholic	35	7.9
	Muslim	20	4.5
	Others**	41	9.3
Marital status	Married	232	52.5
	Single	151	34.2
	Widowed	27	6.1
	Divorced	32	7.2
Level of education	No formal education	53	12
	Primary	148	33.5
	Secondary	133	30.1
	College and above	108	24.4
Occupation	Students	164	37.1
	Housewife	141	31.9
	Government employ	65	14.7
	Merchant	58	13.1
	Others***	14	3.2
Average monthly Income (in Ethiopian *birr*)	Less than 1000	313	70.8
	1000–3000	88	19.9
	Greater than 3000	41	9.3

Others

* = Amhara, Oromo **others**

** = traditional, no religion **others**

*** = no occupation, working in NGO

### Knowledge of respondents about cervical cancer and its screening services

Half of the study participants, 222(50.2%), had good knowledge about cervical cancer screening. The majority of the respondents, 321(72.6%), had heard about cervical cancer, of whom 231(52.2%) had heard about cervical cancer screening. However, only one in ten (10.1%) respondents knew the frequency of cervical cancer screening (Table 2).

**Table 2:** Knowledge of females (15–49 years) about cervical cancer and its screening service in Durame Town, SNNPR, Ethiopia, 2020 (n = 442).

Variables		Frequency	Percent
Heard about cervical cancer	Yes	321	72.6
	No	121	27.4
Heard about cervical cancer screening (n = 321)	Yes	231	52.3
	No	211	47.7
Know the Frequency of screening for precancerous cervical lesions	Yes	45	10.1
	No	397	89.9
Know about sign and symptoms of cervical cancer	Yes	153	34.6
	No	289	65.4
Sign and symptoms(n = 153)	Vaginal bleeding	143	93.45
	Post-coital bleeding	72	47.05
	Foul-smelling vaginal discharge	17	11.1
	Painful coitus	93	6.07
	Others	31	20.3
Know Cervical cancer is communicable	Yes	160	36.2
	No	282	63.8
Know about the risk factors for cervical cancer	Yes	164	37.1
	No	278	62.9
Risk factors for cervical cancer (n = 164)	Family history	148	90.24
	Multiple sexual practices	104	63.4
	Smoking	6	3.7
	STI	38	23.17
	Oral Contraceptive use	17	10.4
	Early sexual practice	128	78.05
	Others	68	41.5
Know the capability of screening to detect early cervical changes	Yes	255	57.7
	No	187	42.3

### Attitude about cervical cancer and its screening service

**Fig 1:**
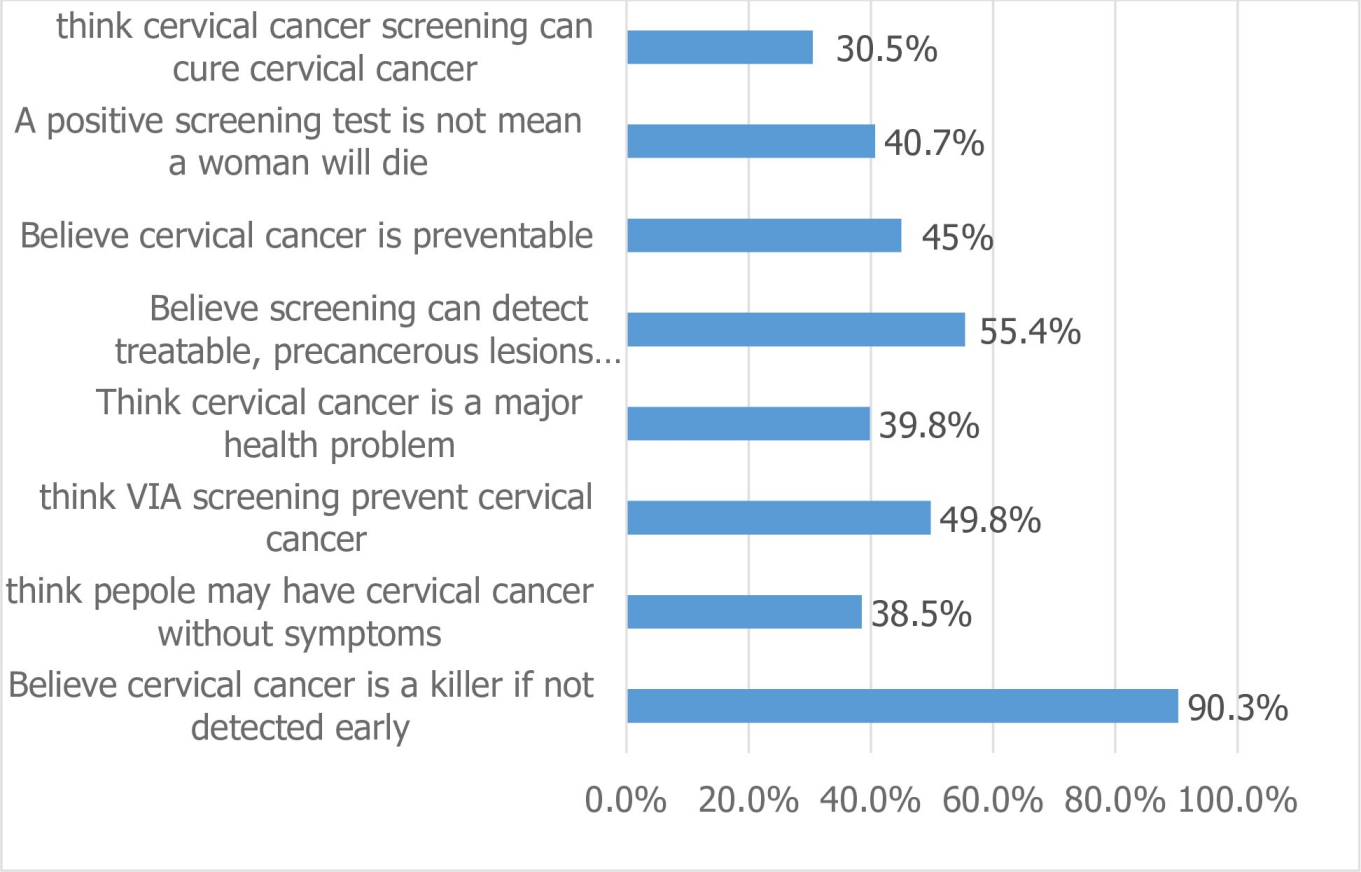
Attitude of females (15–49 years) about cervical cancer and its screening service among in Durame town, SNNPR, Ethiopia, 2020(n = 442).

We found that the majority of the study participants, 236(53.4%), had a positive attitude toward cervical cancer screening. Half of the respondents, 220(49.8%), believed that cervical cancer screening would prevent cervical cancer, and the majority of the respondents, 307(69.5%), believed that cervical cancer screening would not cure cervical cancer (Fig 1).

### Information and invitation about cervical cancer and its screening service by health care providers

Three hundred and fifty-three (80.5%) of the respondents had visited health institutions. Of these, 91(25.6%) of them were informed by health professionals about cervical cancer and the availability of the screening service. Three in ten respondents (29.6%) knew a person suffering from cervical cancer (Table 3).

**Table 3:** Information and invitation about cervical cancer and its screening practice by health care providers among females (15–49 years) in Durame town, SNNPR, Ethiopia, 2020 (n = 442).

Variables	Category	Frequency	Percent
Frequency of your visit to a health institution	Once a year or more	313	70.8
	Once every two years or more	43	9.7
	No visit within the past 3 years	86	19.5
Healthcare provider told you about pre cervical cancer screening(n = 356)	Yes	91	25.6
	No	265	74.4
Ever encountered a person with cervical cancer	Yes	131	29.6
	No	311	70.4

### Sexual and reproductive health characteristics

**Fig 2:**
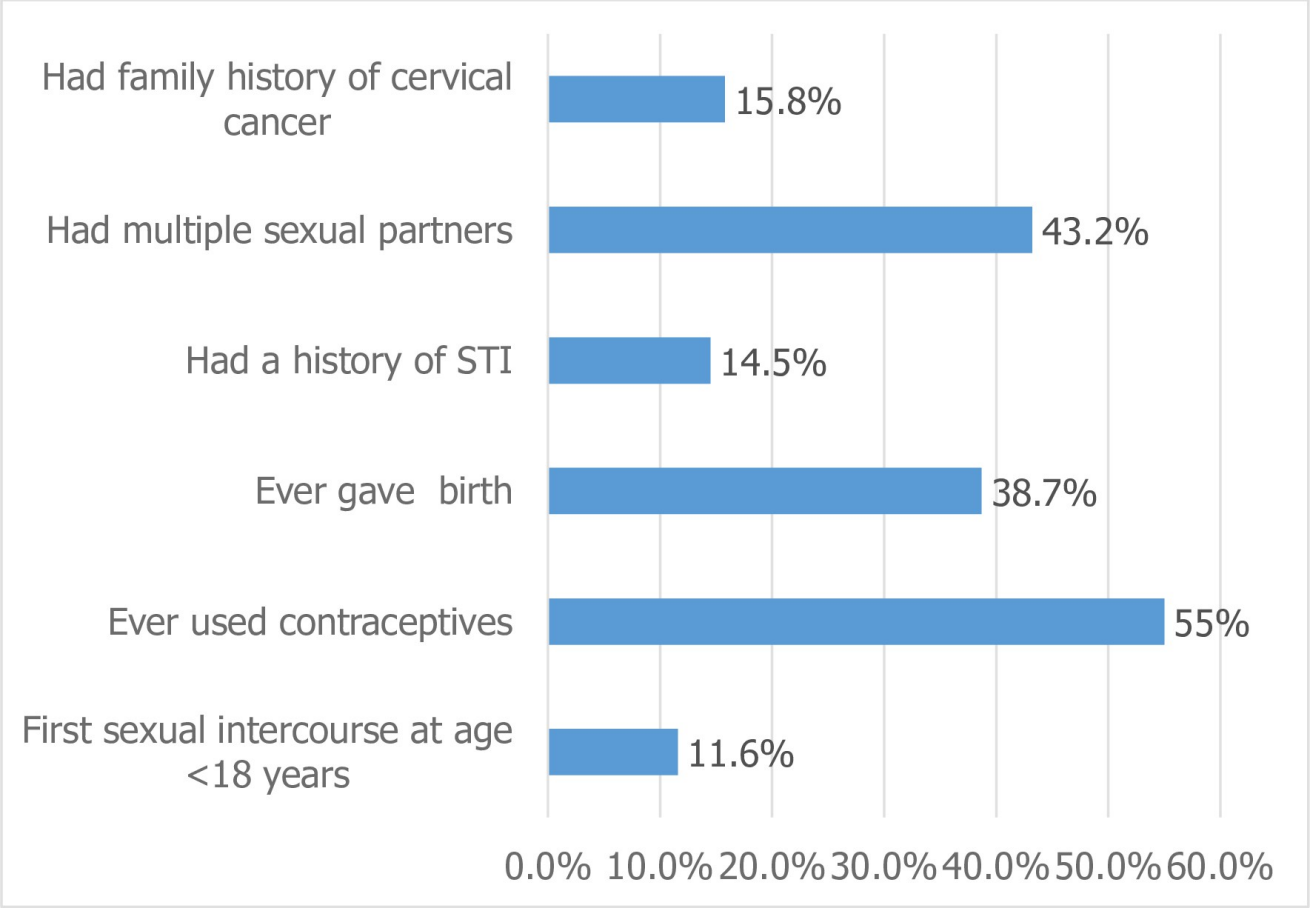
Reproductive health-related characteristics of females (15–49 years) in Durame town, SNNPR, Ethiopia, 2020 (n = 442) (Multiple responses were possible).

We found that 51(11.6%) of females had sexual intercourse for the first time at the age of less than eighteen years. More than half of the respondents, 243(55.0%), had a history of contraceptive use, and 171 (38.7%) had given birth at least once. From the respondents, 179 (43.2%), 62(14.4%), and 70(15.8%) had a history of multiple sexual partners, STIs, and family cervical cancer, respectively (Fig 2).

### Cervical cancer screening practice

**Fig 3:**
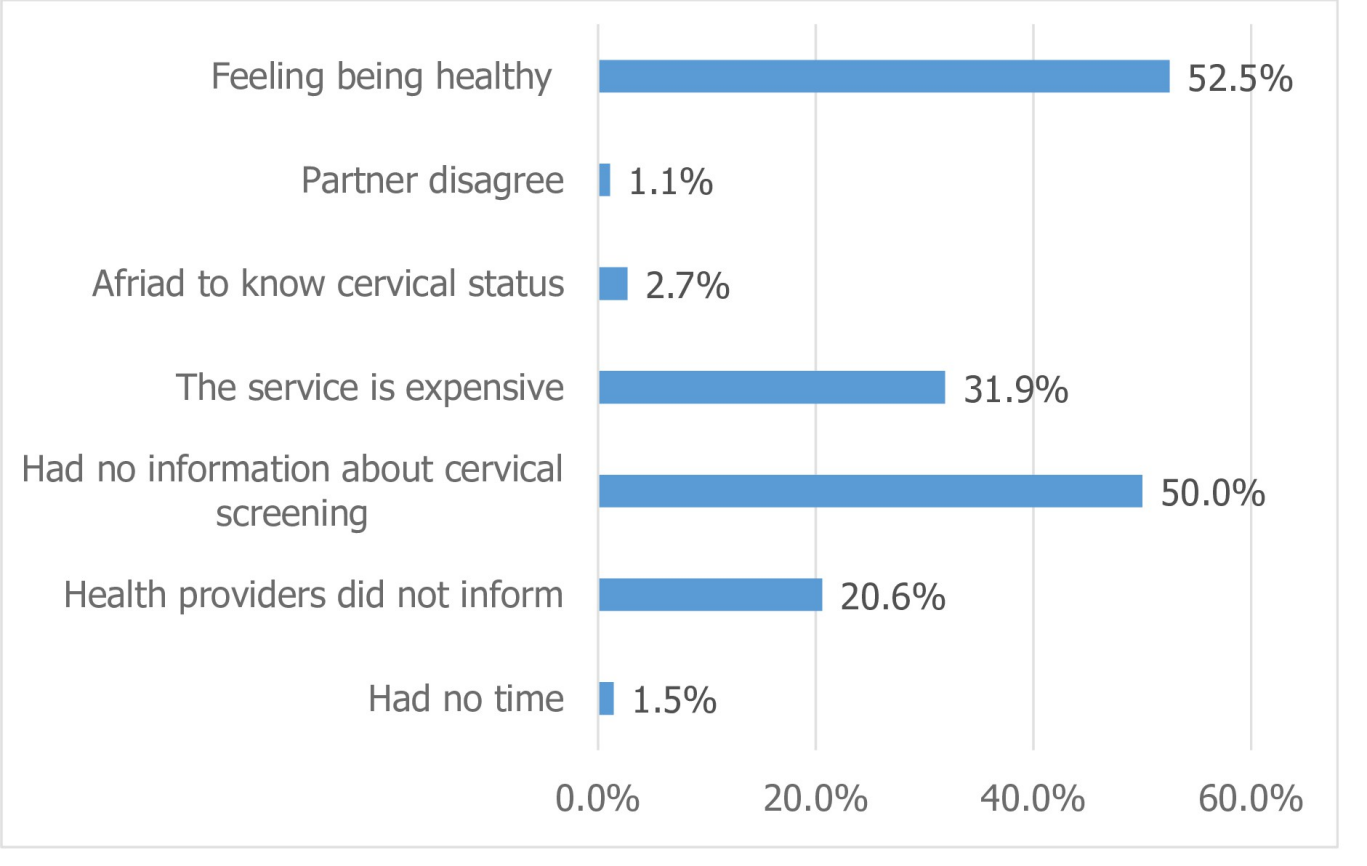
Respondent’s reasons for not utilizing cervical cancer screening service among females (15–49 years) in Durame town, SNNPR, Ethiopia, 2020 (n = 379).

In this study, the magnitude of cervical cancer screening practices was 61(13.8%), with a 95% confidence interval of 10.4%-17.2%. The main reasons for not screening for cervical cancer were feelings of being healthy (52.5%) and a lack of knowledge about cervical cancer (50%) (Fig 3).

### Factors associated with cervical cancer screening practice

Both bivariable and multivariable binary logistic regression analyses were done to identify the factors associated with cervical cancer service utilization. In the bivariable logistic regression analysis, age, monthly income, occupation, educational status, contraceptive usage, family history of cervical cancer, getting information from a health professional about cervical cancer, knowledge, attitude, history of sexually transmitted infections, knowing someone who has experienced cervical cancer, and number of sexual partners were factors that had a significant association with cervical cancer screening practice at a p-value of ≤ 0.25 and were then included in the multivariable logistic regression analysis.

In multivariable logistic regression analysis, the odds of cervical cancer screening practice were 5.4 times [AOR = 5.40, 95% CI: (1.5, 19.5)] higher among those who had good knowledge and 5.2 times [AOR = 5.2, 95% CI: (1.4, 20.0)] higher among those who had a good attitude compared to their counterparts. Those informed about cervical cancer screening by health professionals were 3.58 times [AOR = 3.58, 95% CI (1.3, 9.80)] more likely to practice cervical cancer screening. Similarly, women with a history of STI had 4.2 times [AOR = 4.2, 95% CI (1.4, 12.85)] higher odds of practicing cervical cancer screening than those without a history of STI. Meanwhile, the odds of cervical cancer screening practice were 4.5 times [AOR = 4.5, 95% CI (1.079, 19.051)] higher among those who had an average monthly income of greater than 3,000 ETB than those who had an average monthly income of less than 1000 ETB (Table 4).

**Table 4:** Factors associated with cervical cancer screening practice among females of the reproductive age in Durame town, Kembata Tembaro zone, SNNPR, Ethiopia, 2020.

Variables	Category	Screening service utilization status		Crude Odds Ratio (COR) 95% CI	Adjusted Odds Ratio (AOR) 95% CI
		Utilized n (%)	Non utilized n (%)		
Age in years	15–24	6(4.1)	139(95.9)	1	1
	25–34	34(18.2)	153(81.8)	5.1(2.0,12.6)*	1.2(0.2,4.8)
	35–49	21(19.1)	89(80.9)	5.4(2.2,14.0)*	5.8(0.8,42.0)
Occupational status	Student	6(3.7)	158(96.3)	2.2(0.8,6.1)	0.01(0.0,0.1)
	Housewife	11(7.8)	130(92.2)	15.4(5.9,40.0)*	0.02(0.0,0.2)
	Government employee	24(36.9)	41(63.1)	9.1(3.3,25.0)*	0.2(0.01,3.1)
	Merchant	15(25.9)	43(74.1)	4.6(3.7,57.2)*	0.6(0.07,5.7)
	Others	5(35.7)	9(64.3)	1	1
Average monthly income in Ethiopian Birr	<1000 Birr	29(9.3)	284(90.7)	1	1
	1000–3000 Birr	11(12.5)	77(87.5)	1.3(0.6,2.9)	1.0(0.2,5.6)
	>3000 Birr	21(51.2)	20(48.8)	10.2(4.9,21.1)*	4.9(1.1,22.0)*
Educational status	No formal education	5(9.4)	48(90.6)	1	1
	Primary	10(6.8)	138(93.2)	0.6(0.2,2.1)	3.0(0.2,33.8)
	Secondary	10(7.5)	123(92.5)	0.7(0.2,2.4)	3.0(0.2,35.1)
	College and above	36(33.3)	72(66.6)	4.8(1.7.13.1)*	6.4(0.4,91.7)
Ever used contraceptive	Yes	40(17.7)	186(82.3)	1.9(1.1,3.5) *	1.8(0.6,5.3)
	No	21(9.7)	195(90.3)	1	1
Family history of cervical cancer	Yes	39(10.5)	333(89.5)	3.9(2.1,7.1) *	1.2(0.3,4.3)
	No	22(31.4)	48(68.6)	1	1
Got information from a Health professional about cervical cancer	Yes	42(46.2)	49(53.8)	14.9(8.0,27.8) *	3.5(1.3,9.8) **
	No	19(5.4)	332(94.6)	1	1
Knowledge about cervical cancer and its screening service	Poor	7(3.2)	213(96.8)	1	1
	Good	54(24.3)	168(75.7)	9.7(4.3,22.0)*	5.4(1.5,19.5) **
Attitude about cervical cancer and its screening service	Poor	10(4.8)	196(95.2)	1	1
	Good	51(21.6)	185(78.4)	5.4(2.6,10.9)*	5.2(1.4,20.0) *
History of sexually transmitted infection	Yes	30(48.4)	32(51.6)	10.5(5.6,19.5)*	4.2(1.4,12.8) *
	No	31(8.16)	349(91.8)	1	1
Know someone who suffered from cervical cancer	Yes	39(29.8)	92(70.2)	5.5(3.1,9.8)*	0.6(0.2,1.7)
	No	22(7.1)	289(92.9)	1	1
A lifetime number of sexual partners	Single	14(7.8)	165(92.2)	1	1
	Multiple	47(17.8)	216(82.1)	2.5(1.3,4.8)*	0.7(0.2,2.6)

N.B

* Statistical significance at P<0.05

**Statistical significance at P<0.01

## Discussions

The magnitude of cervical cancer screening practice was 13.8% [95% CI: 10.4–17.2]. A positive attitude, good knowledge, being informed by health professionals about cervical cancer screening, an average monthly income greater than 3000 ETB, and having a history of sexually transmitted infections were factors associated with screening practice.

In this study, the cervical cancer screening practice was 61(13.8%). This finding is in line with previous studies in the country, which revealed the level to range from 3% to 19.8% [16, 20, 23–25]. Whereas the current finding is higher than what had been reported for Nigeria’s 8% [12]. This discrepancy could be explained by the difference in sociodemographic status of the study participants. This is due to the fact that people’s educational and employment status frequently impacts their level of awareness of a particular health condition and their financial ability to seek healthcare services [12]. Moreover, there were differences in the availability of screening services (mostly available in tertiary health institutions) in the Nigerian study [12].

The finding of this study (13%) is lower than studies conducted in Morocco (94%) [26], Thailand (64.9%) [27], Brazil (73–94%) [6], and Australia (82%) [6]. This disparity could be explained by disparities in the study setting and sociodemographic differences among participants. For instance, the study conducted in Morocco was done among women who routinely attend health care facilities, which could increase the chance of screening practices. In addition, the study conducted in Thailand included only females with an age range of 30–60 years, which again may increase the screening practice. Moreover, differences in the health policies of the countries and socio-cultural backgrounds, such as the level of education and health-seeking behavior of study participants, could potentially account for the inconsistency.

In this study, an average monthly income level greater than 3000 Ethiopian Birr (ETB) was one of the factors associated with cervical cancer screening service utilization. This finding is in line with a study done in Arba Minch, Ethiopia [14]. One probable explanation is that, in general, higher income encourages people to seek out comprehensive health and well-being services. Having a good income may also make health facilities more accessible. In addition, since health insurance in Ethiopia is out-of- pocket, service users should cover their health care expenses out of their own pockets. So the monthly income of an individual has an effect on their access to and utilization of health care services. Moreover, it is a fact that individuals with a better monthly income give better focus to their health and seek comprehensive health care, including screening. This could lead to an increase in their practices related to screening.

Women who had a history of sexually transmitted diseases were about four times more likely than those without a history to practice cervical cancer screening. This finding is consistent with the studies done in Ethiopia [20], Zambia [28], and Botswana [17]. One possible explanation is the strongest link that exists between sexually transmitted illnesses and their symptoms. The more STIs a woman has, the more likely she is to become infected with a human immunodeficiency virus, such as the human papillomavirus, which is the most prevalent risk factor for cervical cancer. So, women with STI symptoms may visit health facilities and obtain information about cervical cancer, which may encourage them to seek cervical cancer screening.

Women with good knowledge of cervical cancer and its screening were five times more likely to practice screening. This finding is in accordance with studies conducted in Ethiopia [20, 24], Tanzania [29], and Malaysia [18]. which can be explained by the fact that as women’s understanding of cervical cancer and cervical cancer screening increases, they are better able to balance the risks and benefits of utilizing the service, and their desire to utilize it grows as well. Additionally, because they are aware of the benefits of screening to improve early diagnosis and treatment of cervical cancer, women who have good knowledge of cervical cancer and its screening services are better able to decide whether or not to use it.

In this study, women who had a good attitude were five times more likely to practice cervical cancer screening than those who had a poor attitude. This finding is also in line with reports in Ethiopia [20] and Nigeria [12]. This might be explained by the relationship between attitude and practice, as one of the factors that leads to practice is a positive attitude, which is explained by the health belief model as well [20]. As a result, when women’s attitudes toward cervical cancer screening are positive, the practice of cervical cancer screening increases. As a result, women are more likely to get screened for cervical cancer when they have a positive attitude toward the procedure.

Women who were informed by a health professional about cervical cancer and cervical cancer screening were three times more likely to practice cervical cancer screening than those who were not informed by health professionals. Similar findings were reported from Malaysia [18]. This could be explained by women discussing various health problems with a health practitioner. Reproductive health components account for the majority of them, with the client receiving information on cervical cancer and cervical cancer screening. This prompts them practicing cervical cancer screening. Other researchers have emphasized the importance of providing women with adequate cervical cancer information; they reported that insufficient information provided by healthcare personnel was significantly associated with low Pap smear screening uptake among Malaysian secondary school teachers. Moreover, the Cancer Screening Uptake Expert Panel recently suggested using small media and one-on-one teaching to enhance the uptake of cervical cancer screening [18].

### Strength and limitation of the study

As a strength, this study is a community-based study. Because it is a cross-sectional study, a causal relationship between women’s screening practices and its predictors cannot be established, which could be a limitation of this study.

## Conclusions

Cervical cancer screening practice was very low. Women attitude, women knowledge, acquiring information from health experts, average monthly income, and a history of sexually transmitted illnesses were factors associated with cervical cancer screening practice. Thus, it is necessary to increase women’s awareness, knowledge, and attitude towards cervical cancer and its screening service. In addition, all women who visit a health facility for any reason should be given information about cervical cancer screening by health professionals whenever possible.

## Abbreviations

AOR: Adjusted Odd Ratio
COR: Crude Odd Ratio
DTHB: Durame Town Health Bureau
ETB: Ethiopian Birr
FMOH: Federal Ministry of Health
HPV: Human Papilloma Virus
IHRERC: Institutional Health Research Review Committee
Pap: Papanicolaou
SNNPR: South, Nations, Nationality and People’s Region
STD: Sexual Transmitted Diseases
VIA: Visual Inspection of Cervix by Acetic Acid
VIF: Variance Inflation Factor
VILI: Visual Inspection with Lugol’s Iodine
WHO: World Health Organization
